# What Education Is Doing for Us

**Published:** 1890-09

**Authors:** 


### WHAT EDUCATION IS DOING FOR US.

By comparing modern skulls with those of the same race in an old monastery in the Kedron Valley, Dr. Dight, of the American College of Beirut, Syria, has shown that thirteen centuries have added two inches to the circumference, and three and a half cubic inches to the capacity of the Caucasian skull. The brain has developed in the parts presiding over the moral and intellectual functions, growing higher and longer, without increase of the lower portions, which give breadth to the head, and in which the selfish propensities are centered. This goes to show that brain as well as muscle, is developed by use.

